# Genetic Analyses and Genomic Predictions of Root Rot Resistance in Common Bean Across Trials and Populations

**DOI:** 10.3389/fpls.2021.629221

**Published:** 2021-03-12

**Authors:** Lucy Milena Diaz, Victoria Arredondo, Daniel Ariza-Suarez, Johan Aparicio, Hector Fabio Buendia, Cesar Cajiao, Gloria Mosquera, Stephen E. Beebe, Clare Mugisha Mukankusi, Bodo Raatz

**Affiliations:** ^1^Bean Program, Agrobiodiversity Area, International Center for Tropical Agriculture (CIAT), Cali, Colombia; ^2^Bean Program, Agrobiodiversity Area, International Center for Tropical Agriculture (CIAT), Kampala, Uganda

**Keywords:** genotyping-by-sequencing (GBS), genome-wide association study (GWAS), genomic prediction (GP), root rot disease complex, *Phaseolus vulgaris* L., *Pythium* spp.

## Abstract

Root rot in common bean is a disease that causes serious damage to grain production, particularly in the upland areas of Eastern and Central Africa where significant losses occur in susceptible bean varieties. *Pythium* spp. and *Fusarium* spp. are among the soil pathogens causing the disease. In this study, a panel of 228 lines, named RR for root rot disease, was developed and evaluated in the greenhouse for *Pythium myriotylum* and in a root rot naturally infected field trial for plant vigor, number of plants germinated, and seed weight. The results showed positive and significant correlations between greenhouse and field evaluations, as well as high heritability (0.71–0.94) of evaluated traits. In GWAS analysis no consistent significant marker trait associations for root rot disease traits were observed, indicating the absence of major resistance genes. However, genomic prediction accuracy was found to be high for *Pythium*, plant vigor and related traits. In addition, good predictions of field phenotypes were obtained using the greenhouse derived data as a training population and vice versa. Genomic predictions were evaluated across and within further published data sets on root rots in other panels. *Pythium* and *Fusarium* evaluations carried out in Uganda on the Andean Diversity Panel showed good predictive ability for the root rot response in the RR panel. Genomic prediction is shown to be a promising method to estimate tolerance to *Pythium, Fusarium* and root rot related traits, indicating a quantitative resistance mechanism. Quantitative analyses could be applied to other disease-related traits to capture more genetic diversity with genetic models.

## Introduction

Common bean (*Phaseolus vulgaris* L.) is one of the most important grain legumes for direct human consumption (Broughton et al., 2003). It provides protein, complex carbohydrates, and valuable micronutrients for more than 300 million people in the tropics and 100 million people in Africa alone (Pathania et al., 2014). Common bean production is increasingly affected by root rot diseases, caused by a complex of soil-borne pathogens such as *Rhizoctonia solani*, *Fusarium solani* f. sp. *phaseoli*, *Sclerotium rolfsii*, and *Pythium* spp. (Paparu et al., 2018). Bean yield losses have been attributed to root rot in Eastern and Central Africa where beans are grown extensively (Otsyula et al., 2002; Nzungize et al., 2012). Yield losses of up to 100% have been reported in Uganda, and up to 70% in Rwanda and Kenya (Otsyula et al., 2003); during 1991 and 1993 many farmers stopped growing bean crops due to a serious outbreak of root rot (Nzungize et al., 2012). Disease severity is influenced by several factors, such as poor soil drainage, low fertility, high temperatures and humidity conditions as well as inadequate crop rotation. These environmental factors affect the composition of the root rot disease complex, e.g., in warmer, dryer environments *Sclerotium* may be the major pathogen.

The genera *Pythium*, is considered as an important plant pathogen which attacks multiple crops and is ubiquitous on different continents (Van West et al., 2003). Numerous species of *Pythium* have been recognized as major pathogens, and the most common are *P. aphanidermatum, P. ultimum*, and *P. myriotylum* which are responsible for hypocotyl and root diseases leading to damping off (i.e., plant death during seedling stage) in many crops including dry beans (Abawi and Widmer, 2000; Binagwa et al., 2016). Pieczarka and Abawi (1978) reported that *P. ultimum* acts synergistically with *F. solani f.* sp. *phaseoli* increasing root rot damage to beans, whereas *R. solani* is apparently antagonistic to *P. ultimum*, reducing the severity of root rot. *Pythium* and *Fusarium* spp. were the most frequently isolated fungi in a study in Rwanda and Uganda (Rusuku et al., 1997; Mukankusi et al., 2010), also finding *Macrophomina phaseolina, R. solani*, and *Fusarium oxysporum.* Several studies have reported a higher prevalence of some pathogens inside a complex; for example, Iran, Kenya and Turkey, *F. solani* predominated over *R. solani* and *M. phaseolina* (Erper et al., 2007; Mwang’ombe et al., 2007; Naseri, 2008), while in Michigan (United States), *Fusarium* presented the highest prevalence, followed by *Rhizoctonia.* Additionally, they concluded that the composition of the bean root rot pathogens appears not to depend on the year or the country, because the three main groups of pathogens were consistently recovered (Jacobs et al., 2019). Meanwhile, *Fusarium* and *Pythium* occurred in higher proportions at the CIAT station in Popayán, Colombia (Rojas, 2019).

Identification of beans with genetic resistance to *Pythium* spp. and other root rot diseases is key to allowing breeders to generate farmer preferred cultivars. Consequently, a number of genotypes providing some level of resistance have been identified such as MLB49-89A, MLB48-89A SCAM-80CM/15, and AND1055 (Buruchara and Rusuku, 1992; Mukankusi et al., 2011); RWR2075 and RWR1946 (Buruchara and Kimani, 1999; Namayanja et al., 2014); and RWR719 and AND1062 (Nzungize et al., 2011). Evaluating combined stresses of *Pythium irregulare* with waterlogging in 194 varieties, Navy Veracruz, Negro Argel, and Phavul 77 showed some level of resistance (Li et al., 2016). Moderately resistant varieties to *Fusarium* (G1459, G4795, G5658, Umumbano – G2333, MLB49-89A, Hoima-Kaki, Umgeni and RWR719) and *Rhizoctonia* (PI310668, PI533249) were also reported (Mukankusi et al., 2010; Peña et al., 2013); however, few resistant lines were identified, and new and better resistance sources are required to broaden the genetic base of resistant germplasm for breeders.

Interspecific crosses of *P. vulgaris* with sister species were generated at the CIAT bean breeding program in Cali-Colombia aiming to transfer disease resistance, and abiotic stress tolerance into common bean (Beebe et al., 2011; Butare et al., 2011; Beebe, 2012). A major resistance locus to common bacterial blight was transferred to dry bean from the donor *Phaseolus acutifolius*, resulting in lines with high levels of disease resistance in field evaluations (Singh and Muñoz, 1999). Germplasm derived from *Phaseolus coccineus* × *P. vulgaris* crosses were reported to harbor combined resistance to *F. solani* and *P. ultimum* (Mukankusi et al., 2018). Interspecific crosses are considered to be an option to broaden the genetic base of common bean for their adaptation to different environments.

Several studies investigated the inheritance of resistance to bean root rot. *P. ultimum* was reported to be conditioned by polygenic inheritance or quantitative inheritance (Dickson and Abawi, 1974; Navarro et al., 2008), whereas other studies suggest qualitative single dominant gene in resistance for *P. ultimum* (Otsyula et al., 2003). Ongom et al. (2012) reported single gene resistance for *P. ultimum* and quantitative resistance for *Fusarium solani* in RWR719-derived populations. *Fusarium* and *Pythium* resistances were not related. Mahuku et al. (2005) reported a SCAR marker associated with *Pythium* resistance gene in RWR719 and AND1062 genotypes. Genetics of root rot related traits such as plant emergence and vigor were studied in recombinant inbred line (RIL) populations (Campa et al., 2010), also under flooded and non-flooded conditions (*P. ultimum and P. irregulare)* (Soltani et al., 2018).

Several studies have identified quantitative trait loci (QTL) for *Fusarium* root rot resistance in RILs populations. QTL were identified for *F. solani* (Schneider et al., 2001; Román-Avilés and Kelly, 2005; Hagerty et al., 2015; Kamfwa et al., 2015b), *Fusarium cuneirostrum* (Nakedde et al., 2016) and *Fusarium brasiliense* (Schneider et al., 2001). Even though many studies have been carried out, molecular markers are rarely used in routine breeding. Better resistance sources are desirable, as are markers that work in breeding materials outside the studied RIL populations.

The availability of SNPs markers due to high-throughput genotyping technologies has allowed the development of molecular tools such as Genome-wide association studies (GWAS) and genomic prediction (GP) as support for breeding programs. GWAS aims to detect phenotype to genotype associations to identify major effect genes. GPs are based on a genetic model using genome wide markers simultaneously to estimate genomic breeding values based on the sum of all marker effects. GP is better suited to capture quantitative effects based on many minor genes (Wang et al., 2009).

The objective of the study was to evaluate a panel of lines with interspecific introgressions from *Phaseolus coccineus* for general root rot in the field, and specifically for *Pythium* root rot in the greenhouse. The genetic basis of root rot response was evaluated by GWAS and GP to improve knowledge on the inheritance of *Pythium* root rot resistance and to develop molecular breeding tools that can be deployed in the breeding program.

## Materials and Methods

### Plant Material

A panel of 228 lines (termed RR panel) was evaluated for root rot traits (Table 1). These lines were developed with the pedigrees SAP1-15 × ALB (181×), ABA58 × ALB300-2 (28×) and WAF18 × ALB328 (19×). The ALB lines were obtained from interspecific crosses; *P. vulgaris* × *P. coccineus*, [(SAB516 × (SAB258 × G35066) and (SAB514 × (SAB258 × G35464-5Q)], of which the majority were generated using the parent G35066 and only two using the parent G35464-5Q. F3 individuals were evaluated for root rot-related pathogens in 2014 at Popayan (2°25′N latitude, 76°40′W longitude, 1,730 m.a.s.l.) and 12 of the best and 12 of the least resistant families were selected to generate a population for genetic analysis. Ten individual selections from each family were advanced at the CIAT-Palmira station (3°29′N latitude; 76°21′W, 965 m.a.s.l.), finally resulting in 198 F5 lines, coded as Root rot resistance Andean lines (RRR). Furthermore, 30 bulk-derived lines, coded as Root Rot Andean lines (RRA) were selected for their outstanding performance for root rot resistance (Table 1). MLB49-89A was used as a resistance check for *Pythium*, while GLP2 and CAL96 were used as susceptible checks based on published results (Otsyula et al., 2002). Additional controls of the bean improvement program were used in the field trial (AND1055 and Calima as susceptible; Amadeus as intermediate, and Dicta17 as resistant).

**TABLE 1:** Overview of evaluations and evaluated traits.

		No. Samples*	Control Checks	Traits	Location	Planting Date	Replicates	Generation
**Field**	**Root rot field trial**	198 RRR	Dicta17	NEPl	Popayán	Nov	4	F5
			CAL96	PVg		2015		
				RRD_field				
	**Seed multiplication**	198 RRR	•	100SdW	Palmira	Aug	1	F6
						2016		
**Greenhouse**	**Pyth_E1**	238{180}	AND1055	Pyth	Palmira	Aug	1	F4
		RRR = 136	CAL96			2018		
		RRA = 27	GLP2					
			MLB49-89A					
	**Pyth_E2**	260{220}	AND1055	Pyth	Palmira	Sep	1	F4
		RRR = 186	CAL96			2018		
		RRA = 30	GLP2					
			MLB49-89A					
	**Pyth_E3**	209{204}	AND1055	Pyth	Palmira	Apr	1	F4
		RRR = 195	CAL96			2016		
			GLP2					
			MLB49-89A					
	**Pyth**	•	•	Pyth	Palmira	•	3	F4
**Joined**	**RRD_all**	251{251}	•	Pyth	•	•	•	•
				NEPl				
				PVg				

*n represents the number of samples sown, with the number of used samples indicated in brackets. RRA and RRR used in each trial.

Different data sets previously reported for other common bean root rot-related pathogens in the Andean Diversity Panel ADP were also used in this study. The ADP represents the genetic diversity of cultivated germplasm of the Andean genepool, containing landraces, breeding lines and varieties from public and private breeding programs (Cichy et al., 2015a, b; Kamfwa et al., 2015a; Tock et al., 2017; Oladzad et al., 2019; Zitnick-Anderson et al., 2020). The ADP was evaluated for *Pythium ultimum* (Pyth_UGA) and *Fusarium cuneirostrum* (Fus_UGA) under greenhouse conditions at the CIAT-Kawanda research station (Uganda) as described by Amongi et al. (2020) and Onziga Dramadri et al. (2020). This panel was also evaluated under greenhouse conditions for resistance to *Rhizoctonia solani* (Rhiz_USA) and *Fusarium solani* (Fus_USA) at North Dakota State University (United States) as described by Oladzad et al. (2019) and Zitnick-Anderson et al. (2020). In addition, we used the Angular Leaf Spot (ALS) datasets reported by Nay et al. (2019). These datasets consisted of 316 genotypes (termed extBALSIT panel) which were evaluated under greenhouse and field conditions at multiple sites in Colombia and Uganda.

### Root Rot Phenotyping Under Greenhouse Conditions

A pure colony of a pathogenic isolate of *Pythium myriotylum* preserved on potato carrot agar was reactivated on potato dextrose agar at 24°C for 7 days and then, slants of the pathogen were increased on sterilized sorghum seeds. The inoculum was prepared following the method described by Castellanos et al. (2016). The sterilized soil:sand mix (3:1 ratio) was infected using 0.15 g of inoculum per kg of soil, and then poured into a fiber glass tray (190 × 90 × 25 cm).

Three separate evaluations were performed in the greenhouse at the CIAT-Palmira station (Table 1). On each evaluation, 228 RR lines and 21 checks (15 ALB lines, 4 Andean lines and 2 Mesoamerican lines) were sown in trays containing soil infected with *P. myriotylum*, using rows of 10 seeds per genotype (60 genotypes per tray) following an augmented-block design. The lines MLB49-89A and GLP2 were used as resistant and susceptible controls on each tray. To favor the growth of the inoculum, the soil was watered regularly and the temperature inside the greenhouse was maintained between 24° (night) to 32°C (day). The evaluations of the root hypocotyl and stem were carried out at 7, 14, 21, and 28 days after planting. After the last evaluation, the plants were uprooted to score the root damage following the standard 1 to 9 visual scale developed at CIAT. In this scale, 1 = no visible disease symptoms. 3 = light discoloration either without necrotic lesions or with approximately 10% of the hypocotyl and root tissues covered with lesions, 5 = approximately 25% of the hypocotyl and root tissues covered with lesions but tissues remain firm with deterioration of the root system. Heavy discoloration symptoms may be evident, 7 = approximately 50% of the hypocotyl and root tissues covered with lesions combined with considerable softening, rotting and reduction of the root system. 9 = approximately 75% or more of the hypocotyl and root tissues affected with advanced stages of rotting combined with a severe reduction in the root system (van Schoonhoven and Pastor-Corrales, 1987). The sequential Pythium response (Pyth) scores obtained for the RR panel in the greenhouse were used to calculate the progress of the disease using the area under the disease-progress curve (AUDPC).

### Root Rot Phenotyping in the Field

A total of 216 genotypes, including 198 RR lines and 18 checks (12 ALB lines, 4 Andean and 2 Mesoamerican lines), were evaluated in a field with natural root rot infection at the CIAT-Popayán station (Table 1). Two months before the planting date, the susceptible genotype CAL96 was planted to increase the inoculum and 20 days after germination it was incorporated in the soil. Vegetative material was allowed to break down for 20 days. The trial was planted following an Alpha-Lattice design with four replicates. The experimental units were one-row plots of 2.21 m in length with a row-to-row distance of 0.8 m and 15 seeds per meter. The evaluated variables were: number of plants emerged per complete plot 12 days after sowing (NEPl); three evaluations were conducted for plant vigor (PVg) (12, 35, and 60 days after sowing), using a visual PVg score from 1 to 9 (7 to 9 poor amount of aerial biomass, 4 to 6 acceptable amount of aerial biomass and 1- to 3 excellent amount of aerial biomass). In addition, the weight of 100 seeds (100SdW, g 100 seeds^–1^, weighed on an analytical scale) was evaluated in an unreplicated seed multiplication trial. No destructive sampling was conducted in the field.

### DNA and GBS, SNP Calling

Genotype-by-sequencing (GBS) data for the RR population was generated as described by Gil et al. (2019). DNA was extracted with the Urea-based protocol, according to the method described by Chen et al. (1992) and followed by quality check on 0.8% agarose gels. Quantification was conducted with Quant-iT^TM^ PicoGreen^®^ dsDNA Assay Kit (Invitrogen, Carlsbad, CA), measured in a Synergy H1m (BioTek Instruments, Inc., Winooski, VT, United States). DNA quantity was determined by regression against a standard sample of known DNA concentration. The library for GBS was prepared at CIAT, following the protocol described by Elshire et al. (2011) and sequenced in an Illumina HiSeq 2500 sequencer at the HudsonAlpha Genome Sequencing Center^1^. The ADP accessions were previously genotyped with the same GBS protocol, and the data was retrieved from the ARS-Feed the Future Bean Research Team^2^.

The GBS reads from the RR and ADP populations were mapped to the *Phaseolus vulgaris* reference genome of the accession G19833 (v2.1) (Schmutz et al., 2014). The SNP calling process and VCF filtering was performed using NGSEP (v4.0.3) (Tello et al., 2019), following recommended parameters for GBS data (Perea et al., 2016). The filtering of the VCF removed markers falling in repetitive regions of the genome, as described by Lobaton et al. (2018), genotype call quality below 40, MAF below 0.02, a per-marker heterozygosity rate above 0.02 and markers with less than 285 (∼57%) genotype calls. The filtered VCF contained approx. 20% of missing data that was imputed using BEAGLE (v5.0) (Browning et al., 2018), setting the effective population size at 100 and providing the genetic map reported by Diaz et al. (2020). The genetic position of the final markers was obtained by fitting spline regressions on that genetic map with the R function “smooth.spline” (v4.0.3) with default parameters.

### Statistical Analysis

The AUDPC data from four different evaluations in the greenhouse performed on the RR panel was modeled using the following formula:

(1) $$y_{ijk}=G_{i}+E_{j}+{\left(GE\right)}_{ij}+{\left(ET\right)}_{jk}+\varepsilon_{ijk}$$

Where *y* is a vector of AUDPCs calculated from the four sequential evaluations of Pyth scores, *G_i_* is the effect of the *i*^*th*^ genotype, *E_j_* is the effect of the *j*^*th*^ evaluation, (*GE*)_*ij*_ is the genotype-evaluation interaction term, (*ET*)_*jk*_ is the effect of the *k*^*th*^ tray nested within the *j*^*th*^ evaluation, and ε_*ijk*_ is the error term corresponding to *y*_*ijk*_. In this model, the terms (*GE*)_*ij*_, (*ET*)_*ik*_ were treated as random effects. The term *G_i_*  was treated either as fixed (to calculate best linear unbiased estimators - BLUEs) or random (to obtain an estimate of the genetic variance and to calculate best linear unbiased predictors - BLUPs). Additional models that did not include the evaluation term *E_j_* and its interactions from equation 1 were fitted for each evaluation separately in order to assess their individual variance components, heritabilities, and comparability of BLUEs. We assumed that every random term *u* and the residual ε adjusts to a normal distribution with mean 0 and independent variances *u*~$N\left(0,\sigma_{u}^{2}I\right)$ and ε~$N\left(0,\sigma_{\varepsilon }^{2}I\right)$.

The spatial arrangement of the experiment in the field was used to assign row and column coordinates to each plot. The PVg scores that were obtained from three separate evaluations were modeled using the following formula:

(2) $$\begin{array}{c} y_{ijkl}\\ =G_{i}+E_{j}{\left(GE\right)}_{ij}+{\left(ER\right)}_{jk}+{\left(ERc\right)}_{jkl}+{\left(ERr\right)}_{jkm}+\varepsilon_{ijkl} \end{array}$$

Where *y* is a vector of PVg scores obtained from the field trial, *G_i_* is the effect of the *i*^*th*^ genotype, *E_j_* is the effect of the *j*^*th*^ evaluation, (*GE*)_*ij*_ is the genotype-evaluation interaction term, (*ER*)_*jk*_ is the effect of the *k*^*th*^ replication nested within the *j*^*th*^ evaluation, (*ERc*)_*jkl*_ and (*ERr*)_*jkm*_ are the effects of the *l*^*th*^ column and *m*^*th*^ row nested within the *jk*^*th*^ replication, respectively. In this model, the terms (*GE*)_*ij*_, (*ER*)_*jk*_, (*ERc*)_*jkl*_, and (*ERr*)_*jkm*_ were treated as random effects. The term *G_i_*was treated either as fixed (to calculate BLUEs) or random (to obtain an estimate of the genetic variance and to calculate BLUPs). We assumed every random term *u*~$N\left(0,\sigma_{u}^{2}I\right)$ and the residual ε~$N\left(0,\sigma_{\varepsilon }^{2}I\right)$.

The NEPl and the individual PVg evaluations were modeled fitting a linear mixed model with spatial components using the functions “SpATS” and “PSANOVA” of the R package SpATS (Rodriguez-Alvarez et al., 2020). The phenotypic observations with residuals beyond +/−3 standard deviations from zero in Eqs. 1, 2 and the SpATS model were classified as outliers and removed. The variance explained by the random terms in equations 1 and 2 was tested using the Likelihood Ratio Test (LRT). This value was compared with a chi-square value with one degree of freedom and the significance *p*-value was adjusted following Self and Liang (1987). Broad-sense heritability estimates were calculated using the method proposed by Cullis et al. (2006), using the formula:

(3) H2=1-υBLUP2σ2genotype

Where υ_*BLUP*_ is the mean variance of a difference of two BLUPs of genotypic effects, σ2genotype is the genetic variance.

New variables were also calculated to obtain greater clarity about the general behavior of *Pythium.* The root rot damage score RRD_field is the result of joining the BLUEs of NEPl and PVg traits, assuming that both variables are directly related to the resistance or susceptibility response to the disease in each genotype. These variables were standardized to have mean 0 and variance of 1 before combining them. NEPl was added as a negative value to PVg, so as to obtain a new variable that was positively correlated with the general standard Pyth score proposed by van Schoonhoven and Pastor-Corrales (1987). In addition, an overall root rot damage score RRD_all, combined field and greenhouse traits in the same way. Pearson correlations coefficients between the different traits were calculated, and their significance was tested using a two-tailed *t-*test.

### Genome-Wide Association Analyses for Root Rot Disease

The association analyses for RR were performed using the multi-locus random-SNP-effect Mixed Linear Model tools (mrMLM v4.0), which includes six multi-locus methods (Zhang et al., 2020) and FarmCPU (Liu et al., 2016). The six methods that the mrMLM software includes are: (1) mrMLM, (2) FASTmrMLM (Fast multi-locus random-SNP-effect EMMA), (3) ISIS EM-BLASSO (Iterative Sure Independence Screening EM-Bayesian LASSO), (4) pLARmEB (polygenic-background-control based least angle regression plus empirical Bayes), (5) pKWmEB (polygenic background - control-based Kruskal-Wallis test plus empirical Bayes); and (6) fast mrMLM (FASTmrMLM). The association models account for population structure using the top five principal components as covariates to control for population structure. In mrMLM each marker on the genome is scanned. The Bonferroni correction threshold is replaced by a less stringent selection criterion to identify significant associations. Then, all the markers that are potentially associated with the trait are included in a multi-locus genetic model, their effects are estimated by an empirical Bayes model and all the nonzero effects were further identified by likelihood ratio test for true QTL.

### Genomic Predictions for Root Rot Disease

Genomic prediction (GP) was assessed on each trait of the RR, ADP and extBALSIT panels separately by cross validation, partitioning each panel 50 times randomly into 70% training and 30% validation population. GPs were performed using single Gaussian kernel Bayesian Reproducing Kernel Hilbert Spaces (RKHS) regressions with a fixed bandwidth parameter *h* = 0.5. These predictions were executed using the R package BGLR (v1.0.8) (Perez and de los Campos, 2014) with 10,000 iterations of the sample, 1,000 samples discarded (burn-in) and a thinning factor of 5 to compute posterior means. Prediction ability is expressed as a Pearson correlation coefficient between the observed and predicted breeding values in the RR and ADP panels.

To characterize the influence of the number of SNPs on the GP accuracy of the RR traits, the SNP markers were pruned using two different strategies. The first considered the linkage disequilibrium (LD) between SNP markers. For this, we used the option “-indep-pairwise” of PLINK (v1.90b6.9), with 50 kbp windows, a shift parameter of 5 kbp and maximum pairwise *r*^2^ thresholds of 0.3, 0.5, 0.7, 0.9, and 0.95 (Chang et al., 2015). The second strategy pruned SNP markers randomly throughout the genome.

### Genomic Predictions Between Different Traits and Data Sets

In order to simulate a breeding scenario of employing genomic predictions to select future populations, a pairwise cross-prediction scenario between root rot evaluations on the RR and ADP panels was performed. This scenario was divided in two separate cases. The first consisted in using the data from a given trait (training population) to predict every other trait in the same panel, producing 18 different combinations of training-validation datasets. This case was useful in the RR panel to validate the prediction performance that the greenhouse data can have on the field data, and vice versa. Similarly, this case was useful in the ADP to validate the prediction ability when using data from a root rot pathogen to predict the phenotypic response to another root rot pathogen. The second case used data from a given trait-panel to predict every other trait in a different panel, producing 24 different combinations of training-validation datasets. The second case was useful to explore the prediction ability across populations (ADP-RR), conditions (greenhouse-field) and causal agents of root rots.

## Results

### Root Rot Evaluations in Greenhouse and Field

**FIGURE 1:**
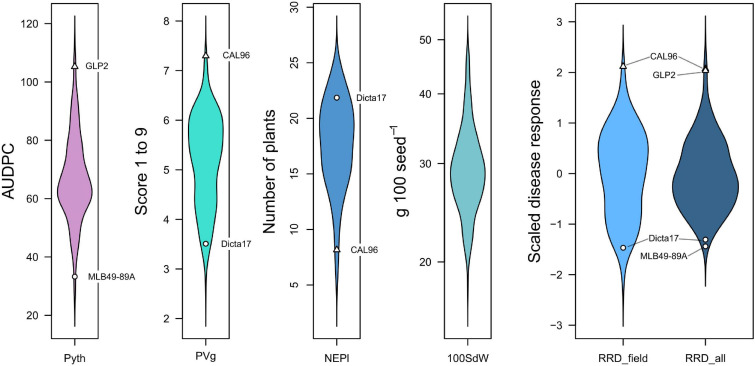
Density distribution of root rot and other traits evaluated in greenhouse and field trials. Resistant checks are marked with circles (MLB49-89A, Dicta17) and triangles indicate susceptible checks (GLP2, CAL96). Traits: Pyth (*P. myriotylum* disease response in the greenhouse as AUDPC), NEPl (number of emerged plants 12 days after sowing in the field), PVg (Plant vigor in the field), RRD_field (root rot damage in the field), RRD_all (root rot damage combining all data sets), and 100SdW (100 seed weight).

In this study we evaluated different symptoms of root rot disease in field experiments, and more specifically *Pythium myriotylum* root rot in the greenhouse. Symptoms included poor seedling establishment/damping-off (NEPl), poor development of aerial biomass (PVg), and root necrosis (Pyth). The phenotypic responses showed normal distributions for most traits in line with a quantitative nature of the disease response (Figure 1). Trait values for positive and negative controls were found as expected on the extremes of the distribution. No clear transgressive segregation for resistance beyond positive checks was observed.

**FIGURE 2:**
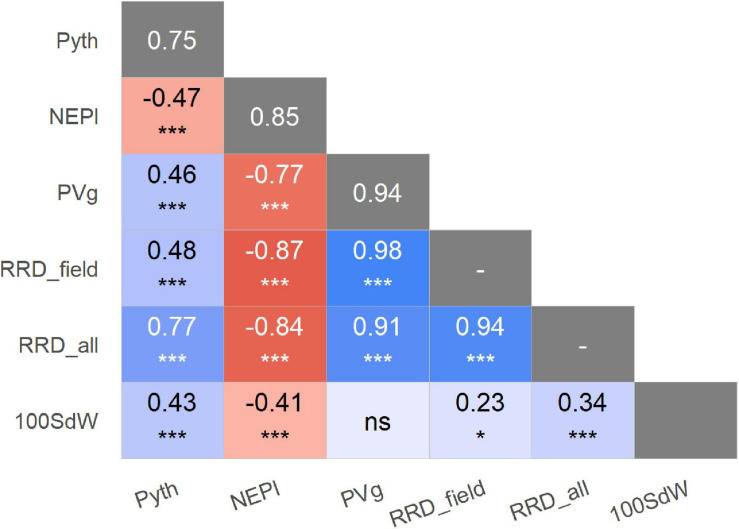
Phenotypic correlations of traits evaluated in greenhouse and field trials, expressed as Pearson coefficients. Significance of correlations indicated as: *p < 0.05; ***p < 0.001; ns not significant. The broad-sense heritabilities are presented in the main diagonal with gray background. Pyth (*P. myriotylum* in greenhouse), NEPl (number of emerged plants 12 days after sowing), PVg (Plant vigor in field), RRD_field (root rot damage), RRD_all (root rot damage combining all data set), and 100SdW (seed weight).

Significant phenotypic variation was detected among lines for Pyth, PVg, and NEPl (Table 2). The genotypic variation was the main source of variability in the trials studied. The estimated broad-sense heritability values were high for all traits (Figure 2 and Supplementary Figure 1); the highest was noted for PVg and NEPl (0.94 and 0.85, respectively). The correlations for Pyth between the three replications over time were significant (0.47–0.56) demonstrating an acceptable level repeatability for these trait evaluations (Supplementary Figure 1) under the presence of significant genotype × evaluation interactions (Table 2). Similarly, Pyth evaluated in the greenhouse showed significant correlations with other disease-related traits (PVg, NEPl, RRD_field, and RRD_all), as well as 100SdW (ranging 0.43–0.77) (Figure 2 and Supplementary Figure 1). This indicates acceptable levels of comparability between the evaluations in the greenhouse and the evaluations in the field. NEPl-PVg correlations were expectedly negative as the traits have inverse scales: resistance to the disease is represented by high NEPl numbers and small PVg scores. The correlation of 100SdW and the disease response may be due to the large seeded Andean parents which are susceptible to root rots. Alternatively, seed size may affect germination speed and susceptibility during the germination phase, which may explain the disease correlation of NEPl but not PVg. Taken together, these results indicate that the Pyth evaluations in the greenhouse and the emergence and vigor evaluations in the field have the same genetic base, and that the same attributes of the lines were evaluated in these quite different trials.

**TABLE 2:** Broad-sense heritability and statistics of the mixed models used to analyze the RR panel data from the field and the greenhouse.

Statistic	Traits		
	Pyth	PVg	NEPl
Heritability	0.75	0.94	0.85
Genotype variance	538.15	1.15	18.55
Gen × Eval variance	279.41	0.046	•
Residual variance	1,077.71	0.62	12.37
Mean	61.45	5.19	17.67
CV	17.54	0.12	18.07
Genotype *p*-value of significance	<2.2E–16	<2.2E–16	<2.2E–16
Gen × Eval *p*-value of significance	<2.2E–16	3.92E–16	•

### Population Structure and Identification of Best Crosses and Lines

**FIGURE 3:**
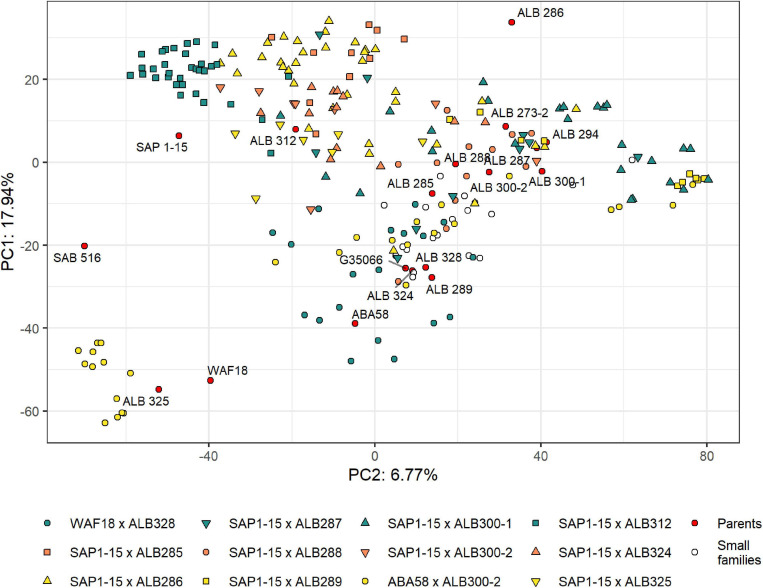
Principal Component Analysis (PCA) displaying the population structure of the RR panel and parental lines. The pedigrees of each larger family (*n* > 5) are represented with colored symbols, smaller families (*n* < 5) are represented with an empty circle. The parents used for the different crosses are located within the graph as red tagged circles.

**FIGURE 4:**
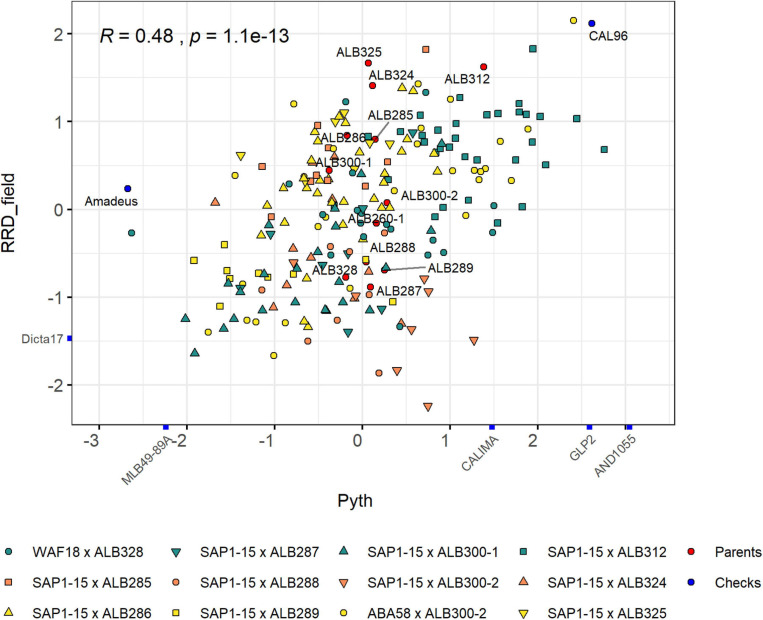
Relationship between *Pythium* damage in the greenhouse and root rot damage in the field for the RR panel. These measurements were scaled to facilitate the comparison. The pedigree of each family is represented with colored symbols. The control checks and the parents used for the different crosses are depicted in blue and red symbols, respectively.

The population structure of the RR panel was evaluated by principal component analysis (PCA) using 15,004 SNP markers. These markers were located mainly on the euchromatic regions of the genome due to the (i) use of the methylation-sensitive enzyme *Ape*KI for the GBS library preparation and (ii) the matrix filter that removed markers from repetitive regions of the genome (Supplementary Figure 2A). However, the genetic scale shows a more uniform distribution of markers along the genome that enriches the regions with higher gene density and recombination frequencies (Supplementary Figure 2B). The first and second PCs explained 24.71% of the variability, revealing moderate but significant population structure (Figure 3). These two PCs mostly separate lines based on their pedigree, with some overlaps. The most separated cluster on the lower left is formed by lines with the pedigree ABA58 × ALB300-2. They originate from a different F2:3 family than the other lines of that same pedigree. During population development, F2:3 families were selected based on their good or bad performance under root rot pressure. This performance is largely mirrored by the advanced RIL lines (Supplementary Figure 3). The best crosses that combine resistance to Pyth in the greenhouse and root rots in the field were SAP1-15 × ALB300-1 and ABA58 × ALB300-2 (Figure 4). The best performing lines were RRR160, RRR161, RRR165, RRR82, RRR84, RRR86, and RRR90, which subsequently have the best score for RRD_all. The susceptible check CAL96 is on the poorest end of the spectrum for both Pyth and RRD_field. The ALB parental lines, however, appear intermediate and not among the best performing lines. Evaluations of parental lines in a previous experiment (Supplementary Table 1) showed *Phaseolus coccineus* ancestors and ALB lines superior to Andean parental lines, however, ALB lines probably underwent unintended selection for root rot tolerance during population advance, interfering with a quantitative comparison of resistance levels or the segregation of potential resistance genes.

### GWAS for Root Rot Damage

GWAS was performed testing 7 different algorithms to identify QTL for root rot disease traits and their associated markers (Supplementary Table 2). These analyses identified possible genomic regions underlying root rot responses; however, numerous inconsistencies between the different models were revealed. We considered SNP markers with significant associations having *p* < 1 × 10^–5^ in two or more models. A QTL located on chromosome 5 at 80,772 bp for PVg, RRD_field and RRD_all stood out explaining 6.86–30.61% of the phenotypic variation. Also, on the start of chromosome 8 four models identified QTL for field root rot traits. However, only one model reported a single significant marker associated with Pyth, showing no overlap with field traits, which would be expected for a major resistance gene. Data for 100 seed weight (100SdW) from the unreplicated seed multiplication trial was evaluated as a control trait for which QTL are usually identified. Five out of seven models identified QTL for this trait at the start of chromosome 7 (100SdW7.1 and 100SdW7.2), presenting the highest LOD identified in 4 out of 7 models. In addition, six models identified other QTL in chromosomes 6 (100SdW6.1) and 8 (100SdW8.1) with lower significance. Identification of strong QTL signals for 100SdW confirmed that this data set is suitable for GWAS; interestingly, not all GWAS models identified these QTL. Taken together, no major resistance genes were found, hence, the results suggest a quantitative inheritance for the observed heritable root rot disease tolerance.

### Genomic Prediction of Root Rot Related Traits and ALS

**FIGURE 5:**
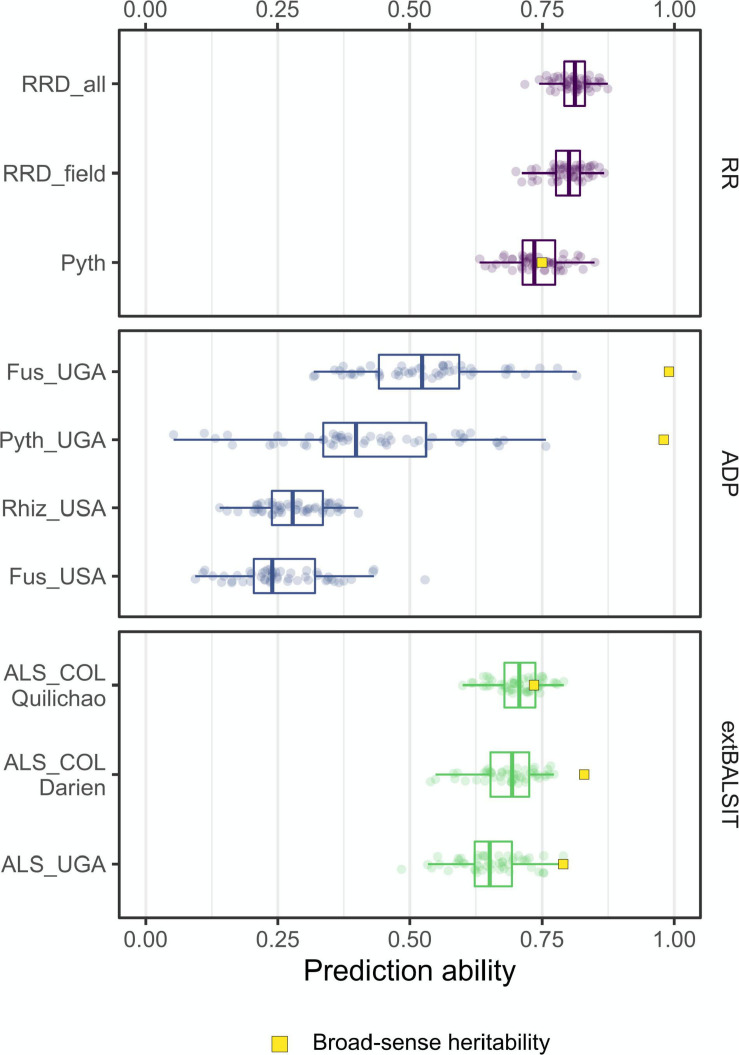
Genomic prediction accuracies for root rot-related traits and Angular Leaf Spot (ALS). Each boxplot shows the distribution of correlation coefficients obtained from the 50-fold cross-validation process. The traits Pyth_COL (*P. myriotylum* response in the greenhouse) RRD_field (root rot damage in the field) and RRD_all (root rot damage combining both datasets) were evaluated in the RR panel in Colombia (COL). The traits Fus_UGA (*F. cuneirostrum*), Pyth_UGA (*P. ultimum*), Rhiz_USA (*R. solani*) and Fus_USA (*F. solani*) were evaluated in the ADP panel in Uganda (UGA) (Amongi et al., 2020; Onziga Dramadri et al., 2020) and the USA (Oladzad et al., 2019; Zitnick-Anderson et al., 2020). The ALS response was evaluated in the extBALSIT panel in Colombia (COL) and Uganda (UGA) (Nay et al., 2019). The broad-sense heritability was calculated using the BLUPs of genotypic effects where they were available.

The accuracy of genomic prediction (GP) models was evaluated using the RKHS model for all traits within each RR trial. In addition, we tested how GP performs in a qualitatively inherited biotic stress trait. GP accuracies of root rot disease traits ranged from 0.7 to 0.8 (Figure 5). The overall root rot disease damage score RRD_all, which incorporates most data, had the highest prediction accuracy (PA). PAs within the RR panel were not significantly affected by the number of SNPs markers, and the data suggests that 1,000–1,500 SNPs is an adequate number to perform predictions in root rot related traits and 100SdW of this study (Supplementary Figure 4). The PAs for the ADP trials were lower, with mean accuracies of 0.42 and 0.53 for the *Pythium* and *Fusarium* trials performed in Uganda (Amongi et al., 2020; Onziga Dramadri et al., 2020), and 0.26 and 0.28 for the *Fusarium* and *Rhizoctonia* trials performed in North Dakota (Oladzad et al., 2019; Zitnick-Anderson et al., 2020; Figure 5). We also evaluated Angular leaf spot (ALS) response data previously published by Nay et al. (2019), who reported a major disease resistance gene by GWAS in a panel of 316 common bean lines. GP accuracies for ALS response in the three locations (Darien, Quilichao and Kawanda) were high, ranging from 0.60 to 0.75 (Figure 5). The GP models explained 0.68, 0.70, and 0.65% of the phenotypic variation for ALS (broad-sense heritability), which is superior to reported GWAS QTL (between 8.6 and 31.4% explained variance) in capturing the genetic variation of this qualitative disease trait.

**FIGURE 6:**
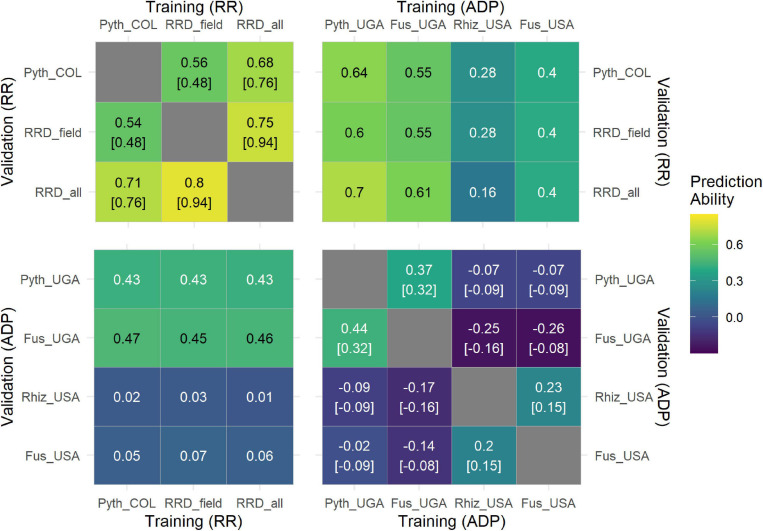
Correlogram with the results of the cross-prediction scenario using different training (X axis) and validation (Y axis) datasets. Predictions within populations are presented in the top-left and bottom-right correlograms. Each value corresponds to the mean correlation coefficient obtained from the 50-fold cross-validation process, while the values in square brackets indicate the phenotypic correlation between each pair of traits. Predictions across populations are presented in the bottom-left and top-right correlograms. Each value corresponds to the correlation coefficient between the observed and predicted breeding values. The cross-prediction scenario was performed for the traits Pyth_COL (*P. myriotylum* response in the greenhouse) RRD_field (root rot damage in the field) and RRD_all (root rot damage combining both datasets) that were evaluated in the RR panel in Colombia (COL). The traits Fus_UGA (*F. cuneirostrum*), Pyth_UGA (*P. ultimum)*, Rhiz_USA (*R. solani*), and Fus_USA (*F. solani*) were evaluated in the ADP panel in Uganda (UGA) (Amongi et al., 2020; Onziga Dramadri et al., 2020) and the USA (Oladzad et al., 2019; Zitnick-Anderson et al., 2020).

To evaluate how GP can predict performance across different evaluations and traits in the RR population, we used RRD_field data to train a GP model and the greenhouse Pyth data as validation set, and vice versa. The average prediction accuracies were similar, field to greenhouse 0.56 and greenhouse to field 0.54, providing a subtle improvement over the phenotypic correlations of 0.48 (Figure 6). Accuracies are expectedly lower than those obtained from internal cross validation procedures. However, they were very close to their observed phenotypic correlations. In summary, prediction accuracies for these data sets of high heritability are strong and the observed highly significant phenotypic correlations between trials are also represented by the genetic modeling across trials.

### Genomic Prediction for Root Rot Traits Across Populations

To investigate a potential general quantitative tolerance to root diseases, we evaluated GP accuracies across previously published data sets on related traits. PAs across trials were investigated with data sets of *Pythium, Fusarium* (Fus), and *Rhizoctonia* (Rhiz) root rot evaluated in the Andean Diversity Panel (ADP) (Oladzad et al., 2019; Amongi et al., 2020; Onziga Dramadri et al., 2020; Zitnick-Anderson et al., 2020). Cross validation within this panel showed highest prediction accuracies for evaluations in Uganda (Pyth_Uga and Fus_Uga) (Figure 6). Evaluating prediction ability across trials, the highest PAs of up to 0.7 were reached using Pyth_UGA data from the ADP evaluated in Uganda to predict RRD_all data in the RR panel in Colombia. Vice versa, the PA was lower (0.43), in line with the much lower genetic variation present in the RR panel, which is an insufficient training set to create a good prediction model for the more diverse ADP. These panels share no common lines. Interestingly, high PAs were also identified across Pyth_UGA and Fus_UGA vs. Pyth_COL data sets, suggesting a common quantitative genetic tolerance for both traits. The genetic correlation evaluated as PA using Fus_UGA and Pyth_UGA as training and validation set, respectively, slightly exceeded the phenotypic correlation between those traits. No high cross prediction ability was found for Rhiz_USA and Fus_USA responses, which also showed low heritabilities and subsequently low PAs within trials.

## Discussion

Soil borne diseases are an increasing constraint for common bean production. Efforts to characterize suitable resistance sources, their genetic inheritance, and development of genetic tools are required to protect bean productivity. Root rot evaluations were often reported to suffer from a moderate heritability, e.g., high sample variation for *F. solani* root rot screening (Schneider et al., 2001). Progress in breeding for field resistance to the root rot complex was hampered by experimental difficulties due to field heterogeneity and large genotype × environment interactions (Beebe et al., 1981; Paez-Garcia et al., 2015). The data set generated in this work in field and greenhouse evaluations looked unimpressive at first, without clear separation of resistant and susceptible genotypes or transgressive segregation. However, the heritability of these trials was high, ranging between 0.71 and 0.94 for root rot related traits, indicating a strong genetic component in the disease response. These results are supported by the suitability of high number of replications, with four replications in the field and three replications over time in the greenhouse.

The strong comparability between field and greenhouse experiments defined by the similarity of their correlations (Figure 2 and Supplementary Figure 2) indicates that the same genetic effects are observed on each trial that was performed for this study. Small scale experiments in the greenhouse should reflect the same disease-related responses in the field (Schneider and Kelly, 2000), as assessments in the greenhouse are often cheaper and allow the evaluation of a large number of samples in a shorter time. The field trial at the Popayan station looked to be affected by root rot to the experienced eye, but it was not clear which pathogen exactly was causing the disease in the trial. A previous collection at this site for fungi and oomycetes identified a large variety of microorganisms, including several *Pythium* species (Rojas, 2019). In spite of that, we observed strong and comparable correlations between agronomic traits assessed in the field and *Pythium* damage evaluated in the greenhouse (Figure 2 and Supplementary Figure 1). In addition, their high heritability indicated a weak influence from environmental factors, contributing to their comparability. The relationship between NEPl and reaction to root rot caused by *P. myriotylum* has been reported by Nzungize et al. (2012). Similarly, plant vigor was reported to be related to *Pythium* (Campa et al., 2010; Soltani et al., 2018), as the plant’s development is affected even if it survives the seedling stage. These results show the consistency between the data from the field and the greenhouse, and the reliability that a combined analysis using both sources of information can provide.

Diverging reports on the genetics of *Pythium-*related resistance exist, stating qualitative responses to *Pythium* root rot controlled by a single dominant gene or the involvement of two or more genes (Otsyula et al., 2003; Mahuku et al., 2005; Nzungize et al., 2012). All the parents of the ALB lines originate from interspecific crosses (*P. vulgaris* × *P. coccineus*), so the initial goal of this study was to identify major resistance genes, based on introgressions from *P. coccineus* (Lobaton et al., 2018). However, ALB lines only showed intermediate resistance. Introgressions from *P. coccineus* in the ALB lines are few due to selection for *P. vulgaris* phenotypes, so *P. coccineus-*derived resistance genes would be expected to follow a more qualitative mode of inheritance. Neither were major resistance genes found in GWAS nor highly resistant lines beyond resistant checks. In spite of this, the dataset used in this study was suitable for association mapping, as GWAS identified strong signals for the control trait 100SdW in chromosomes 6, 7, and 8. The QTL 100SdW6.1 was located at 18,447,838 bp, close to the previously reported QTL 100SdW6.2, identified in the population DOR364 × BAT477 at 20.25 Mbp (Diaz et al., 2017). Several QTL were considered significant by different GWAS models, but no common QTL were identified here for the three evaluated root rot related traits.

Effective genomic cross–predictions of *Pythium* breeding values between the RR panel and the ADP do not support that the observed tolerance is exotic, but rather indicate a general quantitative tolerance to root rots available in Andean germplasm. Predictions across populations have been reported to be poor in lentil (Haile et al., 2020) and other crops (Charmet et al., 2014; Crossa et al., 2014), hence, the effective allelic diversity between ADP and RR panel must be similar. Taken together, our data suggests a quantitative mode of resistance of the traits evaluated here combining favorable different alleles from several precursors, in line with reports on *Fusarium* resistance (Ongom et al., 2012; Wang et al., 2018).

### Genomic Prediction for Root Rot Disease

This study provided GPs for *Pythium* and *Fusarium* root rot disease resistance in common bean in the RR and ADP panels. GP accuracies were high (0.72–0.79 for Pyth and 0.52 for Fus_UGA), in line with the high heritabilities of these traits. GP accuracies of 0.52 and 0.41 were reported in common bean for two types of soybean cyst nematode (Wen et al., 2019). High prediction accuracies have also been reported for the soybean root disease sudden death syndrome (SDS), caused by *Fusarium virguliforme* where GPs accuracies reached 0.64 for root lesions score and lower precision for other root disease related traits (Bao et al., 2015). Prediction accuracies in common bean have been reported to correlate with heritabilities for other agronomic traits (Keller et al., 2020). We observed a similar trend in the RR and extBALSIT populations, but it was not the case for the ADP evaluations in Uganda (Figure 5), where the PA fell by around 40 points below the estimated heritability. This result can be attributable to the binary distribution with presence/absence scores that the original data display (Amongi et al., 2020; Onziga Dramadri et al., 2020), which confers high heritability estimates, and the RKHS model evaluated in this study, which performed the prediction of a trait with quantitative nature. Wen et al. (2019) confirmed that traits with high heritability would have higher PAs. Accuracies generally fall 10–20 points below the heritability of the respective trait, which is similar to most of the observations in this work.

The major molecular breeding application is often considered to be marker assisted selection (MAS), tagging genes with major effects. Genomic selection, on the other hand, has recently been promoted as a genetic tool to predict quantitative traits, yield and yield-related traits being the most investigated. In this work we have seen good GP accuracies for a quantitatively inherited disease trait, hence we extended this analysis to the well characterized qualitative ALS resistance in common bean. We saw that the GP model captures more genetic variation than GWAS in the clear presence of a major resistance gene, which was reported to explain 8.6–31.4% of variation (Nay et al., 2019). In maize, GPs for disease traits were also reported to be superior than GWAS in explaining the phenotypic variance for the quantitative resistance to maize lethal necrosis disease (Gowda et al., 2015). Also in maize, GPs were reported to be of high potential to help improve resistance to *Fusarium* ear rot in the absence of significant SNP-trait associations from GWAS (Holland et al., 2020). Using major loci as co-factors in the linear genomic prediction model has been suggested to improve the model in the presence of major genes. However, we did not observe any advantage to this approach, in line with reports that more often than not these cofactors do not improve model performance (Rice and Lipka, 2019). This suggests that most disease traits may be representable with whole genome models. GP, or its application as genomic selection, requires higher investments in genotyping and analytics compared to MAS, while providing superior information about phenotypic predictions. This apparently also holds true for most disease-related traits, which have often been considered the dominion of MAS. The implementation of genomic selection holds great promise to increase selection precision, allow early generation crosses and to generally select for conditions that the breeder does not have direct access to. This is particularly important for a breeding program such as CIAT’s which is targeting impact regions on other continents. Furthermore, phenotypic results from other panels can be used to train models that can be employed in selection, as we have shown in this study, with good predictability from ADP to RR panel. Especially if genomic models can be generated to allow predictions for several traits at a time, the obtained information may justify the higher investment.

### Genetic Correlation of Resistance Traits

The correlation between field and greenhouse data is not based on a major resistance gene, as one might have expected for a disease resistance. This leaves the question what type of genetically controlled mechanism is conferring the trait correlations. Evaluations of agronomic plant performance in the field and visual scoring disease damage in the greenhouse are very different traits, and environmental conditions are contrasting between the hot greenhouses at Palmira on the one hand, and the Popayan field station at ∼700 m higher altitude on the other. Hence, the similarity in traits cannot just be attributed to adaptation. Also, we observe a strong genetic correlation with *Fusarium* response data from Uganda. Thus, the observed genetic tolerance must be a feature regulated by several genes leading to disease tolerance without being based on a specific resistance gene. Also for ALS, where resistance depends on a major gene, a quantitative model explained more variance, suggesting that other genes have an additional effect on the disease response. GP may capture better the unexplained variance not accounted for by major genes. Cross predictability to other germplasms like the ADP panel and other traits such as *Fusarium* response, indicate that we are looking at a general quantitative tolerance mechanism available in tropical Andean germplasm. This should be further characterized and explored to evaluate if breeding can generate acceptable tolerance levels for farmers following this method.

## Conclusion

Soil borne diseases are a growing threat for bean production, a situation likely to be aggravated by climate change. Hence this trait is of high importance for breeding. Some level of genetic resistance or tolerance was found in this panel, showing good correlations of emergence and vigor observed in field trials with greenhouse data, which should aid selection. Genetic studies revealed no major resistance genes. Instead, genomic predictions performed well, with more potential than GWAS/MAS for these disease traits. We found significant predictive ability across the ADP and the RR panel and also across root rot response data for *Pythium* and *Fusarium* response. Data suggests a general quantitative resistance toward root rot disease present in tropical germplasm. The interactions between various root rot pathogens in bean disease development need further investigation. Applying GS looks promising for semi-quantitative or even qualitative traits, such as disease traits and more. This method needs to be evaluated with other quantitative and qualitative traits, and employed in breeding.

## Data Availability Statement

The SNP marker matrix, the raw and modeled phenotypic data of the RR panel used in this study are available for download at Harvard Dataverse: https://doi.org/10.7910/DVN/SVA5CJ.

## Author Contributions

BR, GM, and SB conceived the study. HFB, VA, and LMD conducted the experiments in the field and greenhouse. LMD, JA, and DA-S performed phenotypic and genotypic data analysis, GWAS, genomic predictions, and interpretation of results. CM provided the ADP data from Uganda. BR assisted in the experimental setup and data analysis. LMD, DA-S, and BR drafted the manuscript. All authors contributed to the article and approved the submitted version.

## Conflict of Interest

The authors declare that the research was conducted in the absence of any commercial or financial relationships that could be construed as a potential conflict of interest.

## Footnotes

1 hudsonalpha.org/, (accessed July 13, 2020).

2 http://arsftfbean.uprm.edu/bean/?p=472, (accessed January 2021).
