# Supplementary material for: Genetic Analyses and Genomic Predictions of Root Rot Resistance in Common Bean Across Trials and Populations
Source: Front Plant Sci. 2021 Mar 12;12:629221. doi: 10.3389/fpls.2021.629221 (PMC7994901; doi:10.3389/fpls.2021.629221)
Supplement: Supplementary file 2 [file Data_Sheet_1.docx]

Supplementary Figures


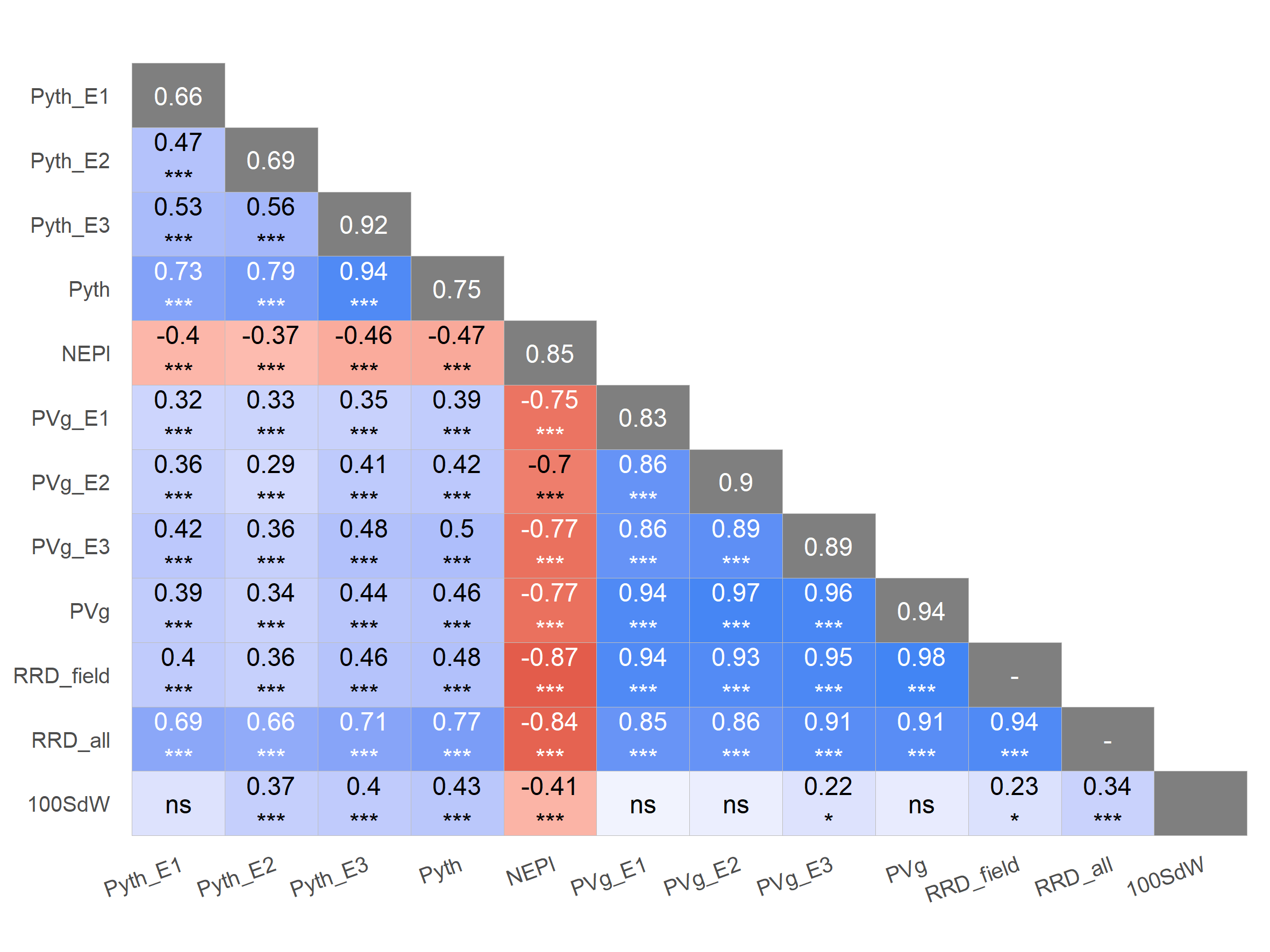
Supplementary Figure 1. Pearson correlations of all traits evaluated in the RR panel. Significance levels of * = p < 0.05, ** = p < 0.01, *** = p < 0.001, ns = not significant. Significant positive correlations depicted in blue, negative correlations in red and heritability in gray color.


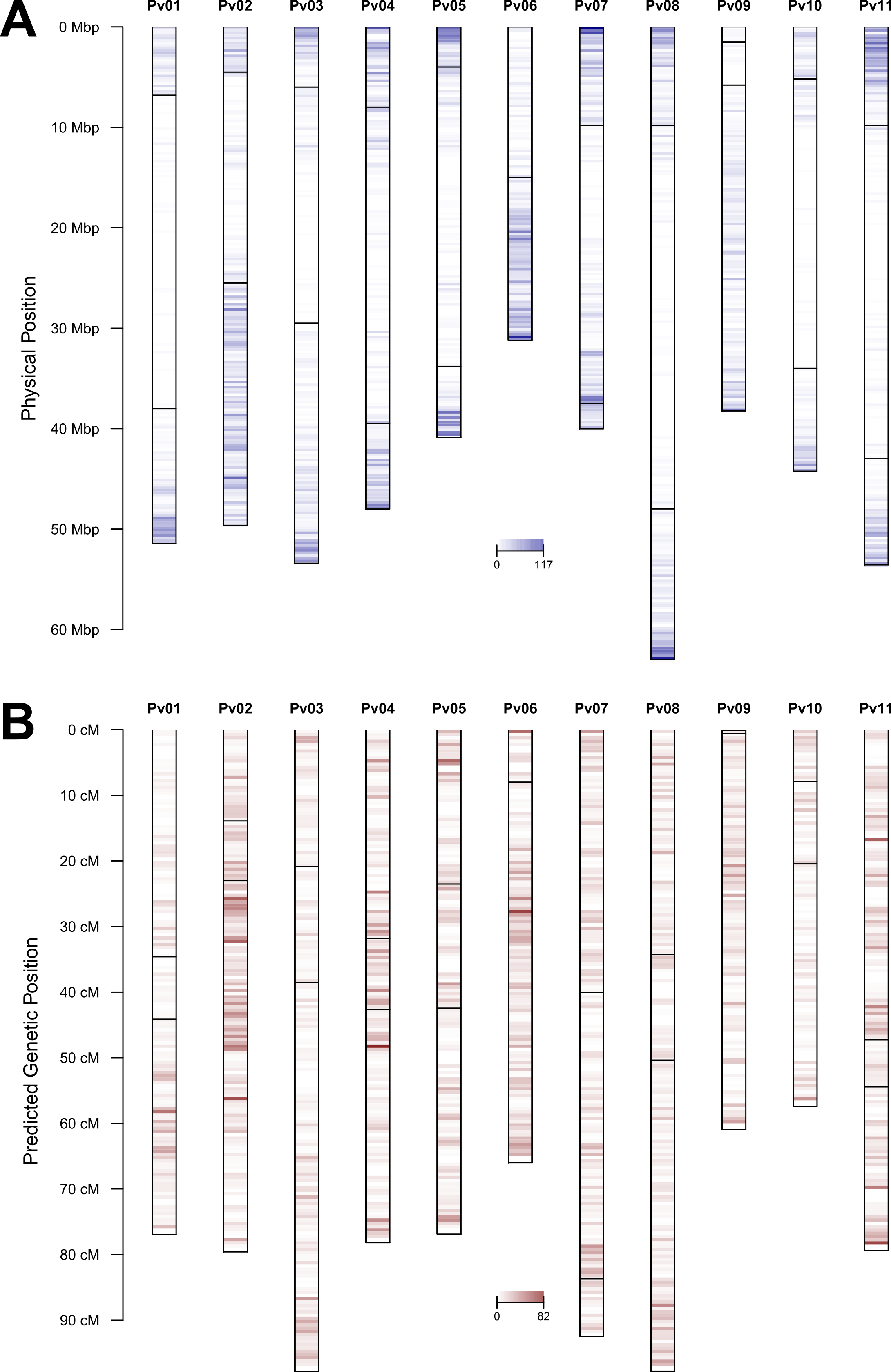


Supplementary Figure 2. Heat map for the density of SNPs along the eleven chromosomes of the *P. vulgaris* reference genome identified by GBS in the RR panel. The heatmap in A) presents the distribution of markers using their physical location. Each color band represents a region of 250 kbp and the color intensity represents the SNP density. The heatmap in B) presents the distribution of markers using their predicted genetic location, which was obtained by fitting a spline regression on the genetic map reported by Diaz et al. (2020). Each color band represents a region of 0.5 cM. The inner black lines on each chromosome represent the boundaries of the pericentromeric regions as defined by Schmutz et al. (2014).


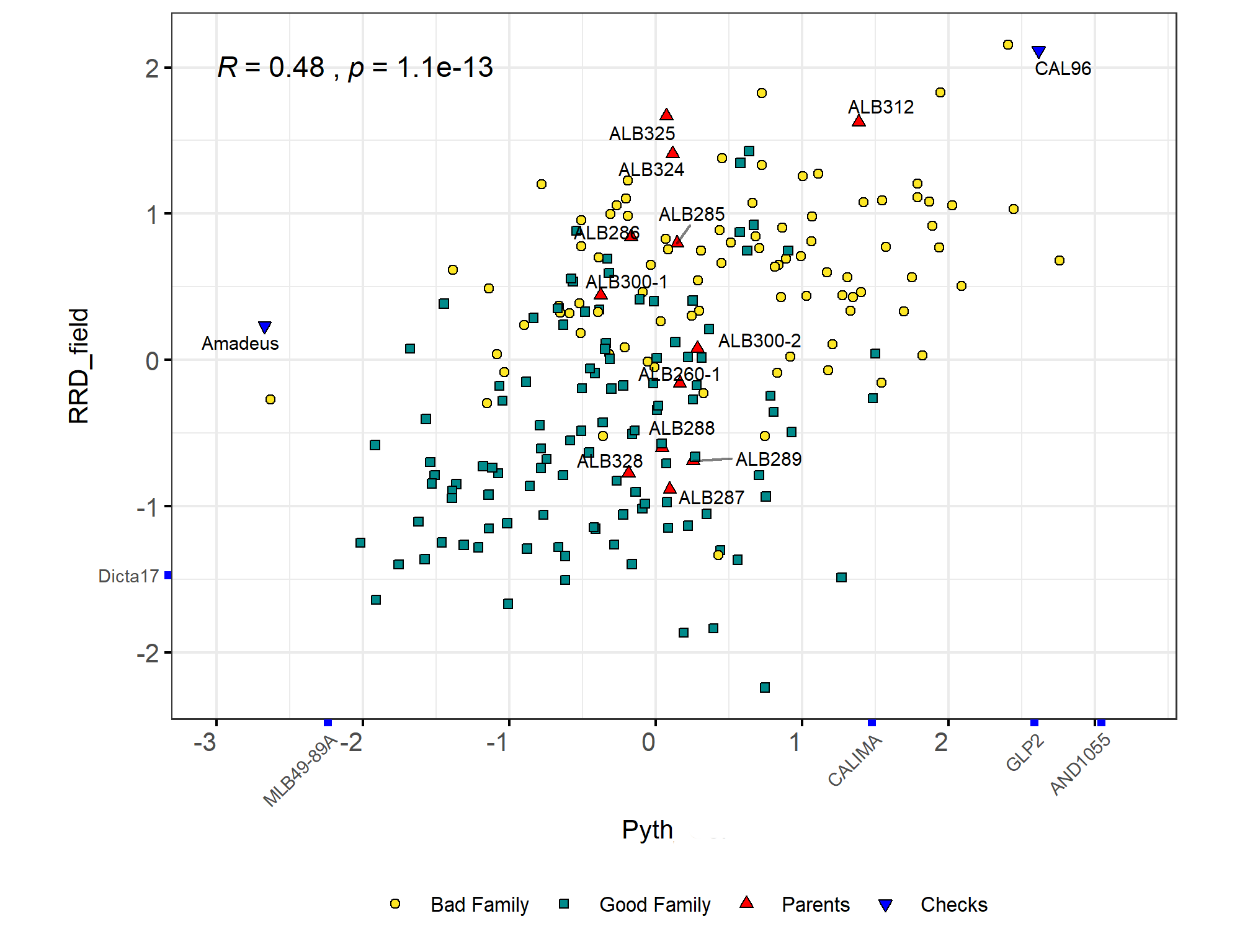


Supplementary Figure 3. Correlation between root rot damage evaluated in Popayan and *Pythium* response evaluated in the greenhouse. Initial classification of good and bad families in respect to root rot damage in the field evaluated in Quilichao in the F3 generation during population development is shown.


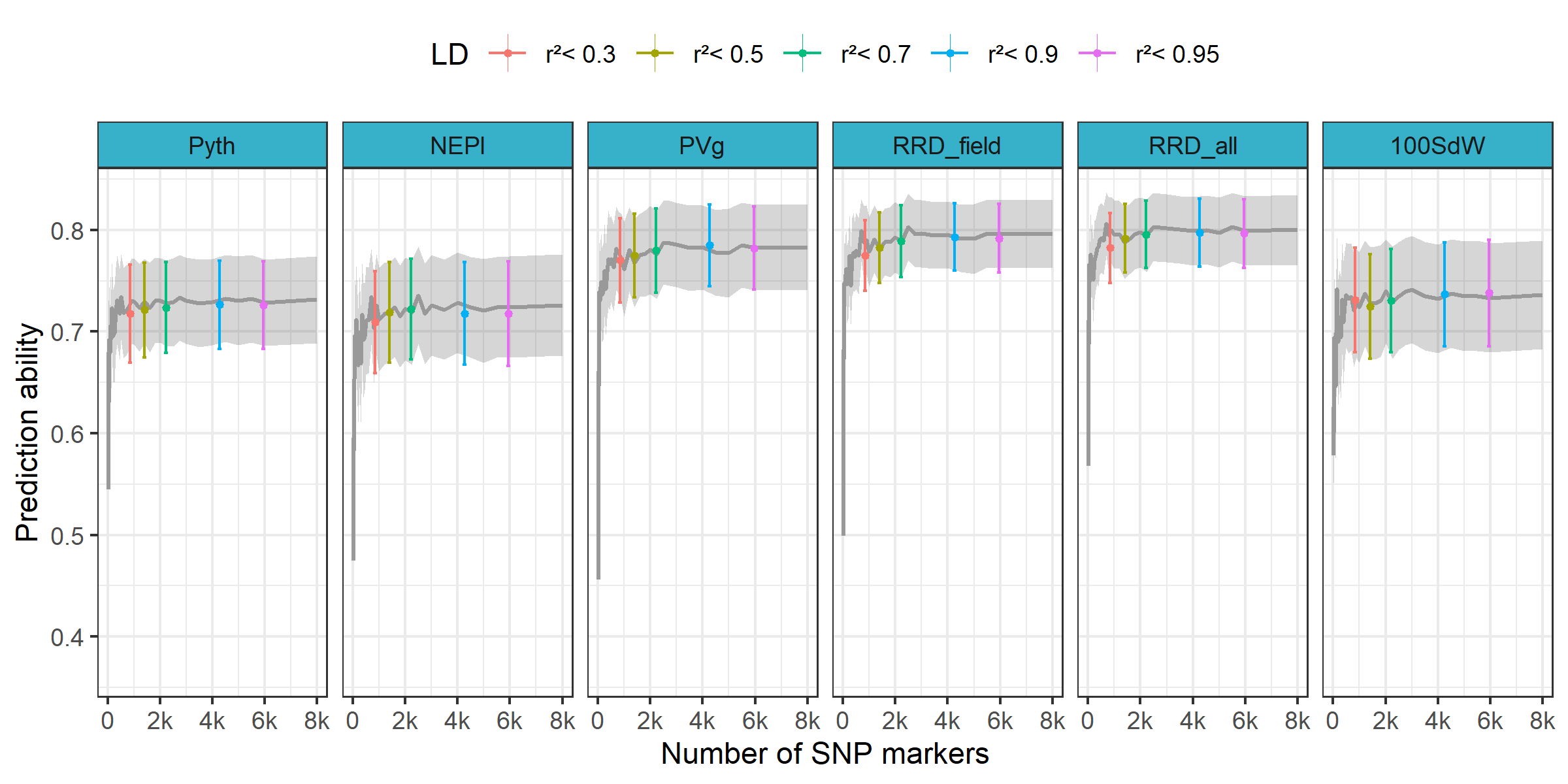


Supplementary Figure 4. Genomic Prediction accuracies in relation to the number of SNP markers in RR panel. The markers used for prediction were chosen either randomly (the gray line and its gray stripe show the average prediction ability and its corresponding standard deviation) or based on variable LD parameters (colored ranges, the middle point and its error bar represent the average prediction ability and its standard deviation). The distribution of values in this plot corresponds to 50-fold cross validations with 70:30 training:validation population partitioning.
